# Influence of Aging on the Physical Properties of Knitted Polymeric Materials

**DOI:** 10.3390/polym16040513

**Published:** 2024-02-14

**Authors:** Antonija Petrov, Ivana Salopek Čubrić, Goran Čubrić

**Affiliations:** 1 Department of Clothing Technology, University of Zagreb Faculty of Textile Technology, Prilaz baruna Filipovića 28 a, 10 000 Zagreb, Croatia; antonija.petrov@ttf.unizg.hr; 2 Department of Textile Design and Management, University of Zagreb Faculty of Textile Technology, Prilaz baruna Filipovića 28 a, 10 000 Zagreb, Croatia; ivana.salopek@ttf.unizg.hr

**Keywords:** polymer, yarn, polyester, recycled, material, aging, measurement, microscopy, roughness, tensile property, water vapor permeability

## Abstract

Nowadays, as consumer expectations have increased worldwide, the importance of polymer materials performance has been raised to a new level. Efforts are required to produce a high-quality product that maintains its quality despite aging factors in certain geographical locations. In the experimental part of this study, polyester materials produced from conventional and recycled yarns, further intended for the production of sportswear, were exposed to natural weathering. Before and after the exposure, the following material properties were investigated: material surface appearance, material thickness, mass per unit area, horizontal and vertical density, surface roughness, tensile properties (force at break, elongation at break), water vapor permeability, liquid dispersion and drying of the material. The results indicate that the surface mass and thickness of all exposed polyester materials decreased after aging due to material shrinkage. The results indicated that prolonged aging negatively affected the values of elongation and force at break. The recycled material exhibited the highest overall decreases in elongation and force at break, but lower surface roughness. In addition, recycled material exhibited a shorter drying time than that of conventional material, both before and after aging.

## 1. Introduction

Polymeric materials age during use, leading to deterioration in performance and an eventual loss of usefulness. Due to aging, the physical-mechanical properties of these materials can be drastically affected when exposed to various influencing factors such as light, heat and water. Recently, researchers from various fields have made considerable efforts to investigate the extent of changes in polymeric materials due to certain exposures [1,2,3,4,5,6,7,8,9,10]. The efforts of scientists have led to the development of various test methods and instruments to accelerate material aging using fluorescent/UV light. These test methods provide a procedure for exposing textile materials of all types to an artificial weathering apparatus under controlled test conditions [11,12,13]. Nevertheless, the methods listed cannot take into account all the specific factors that may contribute to the aging of certain types of products, as may be the case with outdoor weathering. But, as far as outdoor weathering is concerned, the existing guidelines in the international standards [14,15,16] are even more general, as they apply to all types of plastics. They also do not describe the possibilities of taking into account specific aging conditions, such as aging in the sea or in a swimming pool, or segments of human contribution to material aging (human sweating, abrasion of the material during movement, etc.). Therefore, to design an outdoor test, one needs to consider all possible influencing factors and add them to the basic guidelines of the standards. A specific aging protocol may include several variables, including the amount of energy absorbed, the moisture absorbed, the temperature, the duration of exposure to different factors, etc. [17]. To determine how best to age materials and what factors to consider, one must also understand the needs and expectations of the end users of the materials. When it comes to the exposure of materials used in apparel manufacturing, it makes less sense to design a product to perform in all global conditions, as the end product will be overdesigned and less functional. In addition, the cost of material design and production increases significantly in this case. Experience has shown that products can be designed to work under most global conditions but fail under conditions typical of a particular micro-location. Today, as global user expectations have increased and the importance of performance and well-being has been elevated to new levels, efforts are needed to produce a high-quality product that maintains its quality despite aging factors in specific geographic locations.

The degradation of polymers, including polyester in particular, is also a growing concern, due to the accumulation of plastics and microplastics in the environment. The studies conducted so far in this field have focused on different aging protocols and have shown that the exposure of polymers to different environmental conditions affects certain mechanical and physical properties [18,19,20,21,22]. Some researchers have also confirmed the degradation of polymer fibers at the molecular level. Arhant et al. [20], for example, aged polymers at high temperatures (80–100 °C) in water and then analyzed them for their molecular mass, crystallinity ratio and tensile behavior. The results of the study showed a significant decrease in the values of the observed properties. The study, which focused on the degradation of polymers immersed in swimming pool water, showed a decrease in the average diameter of the produced PET material [21]. It was also shown that the degradation of the polymer occurs in the crystalline phase. As for polymers for apparel applications, the aging protocol should consider the regular care process as well as the polymer treatments, in addition to the environmental factors in aging [23,24,25,26]. As reported in studies, the care factors themselves can influence the increase of material strength as a result of polymer fibrillation and material shrinkage [27]. Any changes in the physical-mechanical properties of the polymer material may lead to undesirable changes in the overall perception of comfort, which is nowadays considered a crucial requirement for this group of final products [28]. Due to the ever-growing interest in all sports-related segments, researchers are increasingly interested in studying and improving the properties of materials used in sportswear [29,30,31,32,33,34,35,36,37,38]. However, to the best of the authors’ knowledge, there is a lack of specific protocols for the aging of polymeric materials intended for the production of functional sportswear. In our previous studies, the first steps were taken to close this gap [39,40,41]. As reported in these studies, aging affected the physical-mechanical properties of the material, but not to an extent that would be expected to have a significant negative impact on an athlete’s performance.

The study presented in this paper focuses on the implementation of a redesigned and improved aging protocol for a specific group of polymeric materials—more specifically for materials used in the production of functional sportswear. The protocol includes exposure segments that correspond to the typical exposure of materials worn by a professional athlete. The study analyses representative materials produced from conventional and recycled polyester in terms of their physical properties and observes the changes in their properties due to aging.

## 2. Materials and Methods

### 2.1. Material Selection

Four materials were selected for the study. The selected materials are representative fabrics available on the market that are intended for the manufacturing of football sportswear. The materials were produced as knitted fabrics (i.e., structures consisting of loops as the main unit, repeated in the horizontal and vertical direction). The knitted structures were selected for this study as they provide better body fit and comfort during sports activities. All selected fabrics were made of 100% polyester yarns, both standard and recycled. The fabrics differ in mass per unit area (130–155 g/m^2^, Table 1). An overview of the selected knitted fabrics with the most important details, corresponding material ID, composition, mass per unit area, and yarn fineness, can be found in Table 1.

### 2.2. Aging Protocol

The aging protocol for the selected materials included several key steps and environ-mental conditions. Over a four-week period, the materials were subjected to a simulation of the conditions to which professional football players are exposed during their training regimen. This process took place five days per week. The aging process consisted of two distinct phases, each lasting two hours per day.

During the morning session, which took place from 8:00 a.m. to 10:00 a.m., the materials were exposed to direct sunlight to simulate the conditions of an outdoor training session. After 15 min of exposure, which simulated the onset of physical activity and sweating, an artificial sweat solution was sprayed evenly onto each material. The artificial solution used was the solution of acidic sweat powder with a pH value of 5.5, prepared in accordance with BS EN ISO 105-E04 [42]. This application was repeated ten times to ensure an even coating on both sides of the materials. The materials were then exposed to direct sunlight for an additional two hours until 10:00 a.m. to simulate the effects of strength training, stretching, and warm-up exercises common to professional football players.

The second phase simulated a football game that lasted from 10:00 a.m. to 12:00 p.m. Similar to the first stage, after 10 min of exposure, the same artificial sweat solution was evenly sprayed on each material. After the completion of each daily aging session, including the strength training and football game simulation phases, the materials were cleaned. The cleaning of the polymeric materials included standard washing using detergent without phosphate and optical brighteners. 

The aging simulations were carried out during the summer (month of August) at coordinates 45°48′055.4364″ N and 15°57′059.6448″ E. All data were monitored by the European Meteorological Center ECMWF with the weather forecast model HRES [43]. Throughout these experiments, the average air temperature was 25 ± 2 °C, with an average relative humidity of 66 ± 5%, an average air pressure of 1016 ± 2 hPa, an average wind speed of 4 ± 2 m/s, and an average UV index of 4 ± 2. 

### 2.3. Methods of Measurement 

A series of tests and analyses were carried out on the polymeric materials to evaluate the performance and changes resulting from the simulated aging process. In the experimental part, the following properties of the selected materials were studied: the appearance of the material surface via microscopic analysis, material thickness, mass per unit area, horizontal and vertical density, surface roughness, tensile properties (force at break, elongation at break), water vapor permeability, liquid dispersion, and drying of the material. A FTIR analysis was also conducted.

#### 2.3.1. Microscopic Analysis 

The surface appearance of the materials was visualized using a Dino-Lite Edge AM7915MZT digital microscope (Dino-Lite, Almere, The Netherlands). The microscope is equipped with a 5-megapixel edge sensor and features the EDR (Extended Dynamic Range) function, which can recover details in darker or brighter areas of a specimen by capturing images at different exposure levels. It also has the EDOF (Extended Depth of Field) function, which can automatically stack images at different focus levels to increase depth of field, especially on rough or uneven surfaces. Prior to testing, all samples were cut to a size of 100 × 100 mm under controlled environmental conditions at an air temperature of 20 ± 2 °C and a relative humidity of 60 ± 5%. A 200× magnification was used for microscopic analysis, and Dino Capture 2.0 professional software (Dino-Lite, The Netherlands) was used for image analysis.

#### 2.3.2. Thickness and Mass per Unit Area Testing 

The thickness of the material was measured as the vertical distance between the reference plate on which the specimen was placed, and the parallel circular plate covering the specimen under pressure, as described in ISO 5084 [44]. A thickness gauge DM-2000 (Wolf Messtechnik GmbH, Freiberg, Germany) was used for the test. The specimens were conditioned in a standard atmosphere prior to testing, as described in ISO 139 [45]. During the test, a pressure of 1 kPa was applied to a sample area of 20 cm^2^. A total of 10 measurements were made at different locations on the specimen. The test areas for the thickness measurement were located in the central part of the 20 cm^2^ specimen, and were arranged diagonally (starting from the lower left corner of the sample). The edges of the samples were avoided. The average thickness result was presented as the mean value of 10 measurements. The mass per unit area of polymeric materials was measured using the Kern ALJ 220-4 analytical balance (Kern & Sohn, Balingen, Germany) on samples measuring 100 × 100 mm.

#### 2.3.3. Horizontal and Vertical Density

The measurements of the horizontal and vertical densities were carried out using a Dino-Lite PRO HR digital microscope (Dino-Lite, The Netherlands) together with professional software, under a magnification of 200×. The measurement was performed on a flat surface and samples were cut to 100 × 100 mm in size. The measurement was conducted under controlled conditions, temperature, 20 ± 2 °C, and relative humidity 65 ± 2%. To determine the horizontal loop density (Dh), the samples were analyzed by counting the loops in the course direction at a material length of 10 mm. On the other hand, for the determination of the vertical loop density (Dv), loops were counted in a wale direction at the same material length. This procedure was repeated at 10 different positions along the material surface (for both determinations of the Dh and Dv) to ensure the reliability and accuracy of the density results.

#### 2.3.4. Surface Roughness Testing

The surface roughness tester PCE-RT 2000 (PCE Instruments UK Ltd., Southampton, United Kingdom) was used to determine the surface roughness. The roughness tester works following the touch method in accordance with ISO 3274 [46]. The roughness with this measuring device is precisely recorded via the piezo sensor. To analyze the surface structure and interpret the results, the R_a_ parameter was selected. This parameter represents the average value of the absolute deviations of the height structure of the surface (marked as Z_1_, Z_2_… Z_n_) from its mean line within a specified length. R_a_ was calculated using the following mathematical equation:

(1) $$R_{a}=\frac{\left|Z_{1}\right|+\left|Z_{2}\right|+...+\left|Z_{n}\right|}{n}$$

(2) $$R_{a}=\frac{1}{n}\sum_{i=1}^{n}\left|Z_{i}\right|$$

In this equation, Z_i_ is related to the ordinate values and n is their number. R_a_ represents the average “roughness” or “irregularity” of the material’s surface within a specific segment. The larger the R_a_ value, the rougher or more irregular the material’s surface is. The roughness tester was placed on a surface for the measurement. During the measurement process, a probe was pulled over the surface [47]. The sampling length was set default to 0.80 mm. The surface roughness of the material was measured in three directions of the polymeric material: horizontally, vertically and diagonally.

#### 2.3.5. Tensile Testing

The tensile properties of the materials were tested according to the procedure described in the international standard ISO 13934-1 [48]. The Statimat M tensile tester (Textechno, Germany) was used for the measurements of force at break and elongation at break. The accuracy of the tensile testing device corresponded to a class meaning that the error of the distance of the clamps was ±1 mm. The test was performed using two pneumatically active clamps, of which the upper one was static, and the lower one was attached to a stretching slide. The distance between the clamps was set to 100 mm. The samples were prepared to dimensions of 50 × 200 mm. Prior to testing, specimens were conditioned at the standard test atmosphere, i.e., temperature, 20 ± 2 °C and relative humidity, 65 ± 3%.

#### 2.3.6. Water Vapor Permeability Test

To test the water vapor permeability, the moisture meter PCE-MA (PCE Instruments UK Ltd., United Kingdom) was used. Specimens with dimensions of 100 ± 2 × 100 ± 2 mm were cut and conditioned for 24 h in a room at a temperature of 20 ± 2 °C and a relative humidity of 65 ± 3%, as defined in ISO 139 [45]. The measurements were performed with the installation of a selectively permeable foil under the specimen. During the measurements, the temperature was set at 41 °C and the duration of the test measurement was set at 900 s. The water vapor permeability (W) was calculated according to the following formula:

(3) $$W=\frac{∆m}{A\cdot t\;}$$

where, *W*—is the water vapor permeability (g/m^2^h); ∆*m*—change in mass of the specimen; *A*—the specimen area (m^2^); and *t*—the testing time (h).

#### 2.3.7. Liquid Dispersion and Drying of Material

The TESTO 872 infrared thermal imaging camera (TESTO, Germany) was used to observe the liquid dispersion on the surface of the specimen. Specimens with dimensions of 100 × 100 mm were placed on a flat surface in a room with an air temperature of 20 ± 2 °C and 65 ± 3% relative humidity. A solution of artificial sweat was applied vertically with a pipette at a distance of 20 mm from the surface of the specimen. A thermal imaging camera was used to detect the different phases of fluid transport and capture images, which were then used to measure the corresponding parameters. The experiment focused on the following parameters:

- Wetting time, i.e., the time required for the absorption of the applied solution,
- Wetting surface area, i.e., the surface area of the specimen with absorbed solution. The surface area was determined based on the maximum length of the wetted area in the directions of the x- and y-axes and according to the following formula:

(4) $$A=\frac{x\cdot y\cdot \pi }{4}$$

where: *A*—wetting surface area (mm^2^); *x*—wetting diameter in the direction of the *x*-axis (mm); *y*—wetting diameter in the direction of the *y*-axis (mm).

- Drying time, i.e., the time required for a solution to be absorbed. The time at which the solution was absorbed was defined as the time at which there were no differences between the temperatures of the wetted and non-wetted zones (what was observed trough the thermal image). The number of measurements performed for each polymeric material was 5.

#### 2.3.8. The FTIR Analysis

The FTIR spectra of the polyester materials were recorded with the Perkin Elmer Spectrometer—Spectrum Two (Perkin Elmer, Waltham, MA, USA). A total of 10 scans were performed for each material studied. The spectral resolution was 4 cm^−1^. The scans of materials were performed before and after aging in order to draw conclusions about possible chemical changes in the polymer.

## 3. Results and Discussion

### 3.1. Results of Microscopy Analysis

Table 2 shows microscopic images of selected materials before and after aging. Visual changes that occurred after aging can be seen. The changes in samples M-155 and M-151 after aging are particularly striking. Small additional fibers that are within the fabric structure are clearly visible (additionally highlighted by red circles in the images presented in Table 2). This likely occurred due to the exposure to sunlight and care processes (in this case, material washing). This is in line with the conclusions of Hazlehurst et al. [27] who pointed out that most microfibers are loosely bound in the fabric and yarn structures and detach relatively easily and quickly due to material care.

On samples M-145 and M-130, the changes are not as pronounced, but slight fibers protruding from the fabric structure can be seen. In summary, the microscopic images of the specimens before and after aging reveal visual changes in the material structure, including the presence of additional fibers. The changes are more pronounced in the materials composed of conventional yarns with a higher nominal mass per unit area. The changes were not observed in the material made of recycled yarn (i.e., specimen M-130). This can be explained by the fact that the surface of the recycled yarn was already more compact before aging due to the recycling process so that additional protrusions of the fibers did not occur. The changes described can affect the properties and functionality of the materials over time, which is crucial for understanding the long-term durability and performance of sportswear materials. In addition, these changes can also affect the tactile sensation of the wearer.

### 3.2. Results of Thickness and Mass per Unit Area Testing 

**Figure 1:**
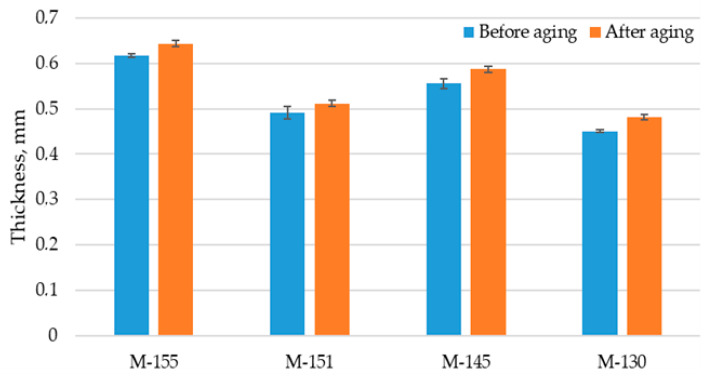
Thicknesses of materials before and after aging.

The values for the thickness of unexposed materials range from 0.45 to 0.62 mm, while those of exposed materials are 0.48–0.64 mm (Table 3). As can be seen from the comparison in Figure 1, the thicknesses of all tested materials increased slightly after aging. These results are reliable due to the very small variation between the repeated values, which was confirmed by the coefficient of variation (0.68–2.79%), as well as the low values of standard deviation (presented in Figure 1). The observed decreases in thickness can be explained by the increased shrinkage of the material as well as by the increased number of particles within the material structure, which was previously discussed based on the microscopic images. It should also be noted that the greatest increase in thickness was observed in the material produced from recycled polyester (M-130), suggesting that the loop structure of the recycled material is less stable when exposed to aging factors, i.e., it has a greater tendency to deform. This behavior is expected to affect various physical-mechanical properties of a material, which will be discussed in the next sections.

**Figure 2:**
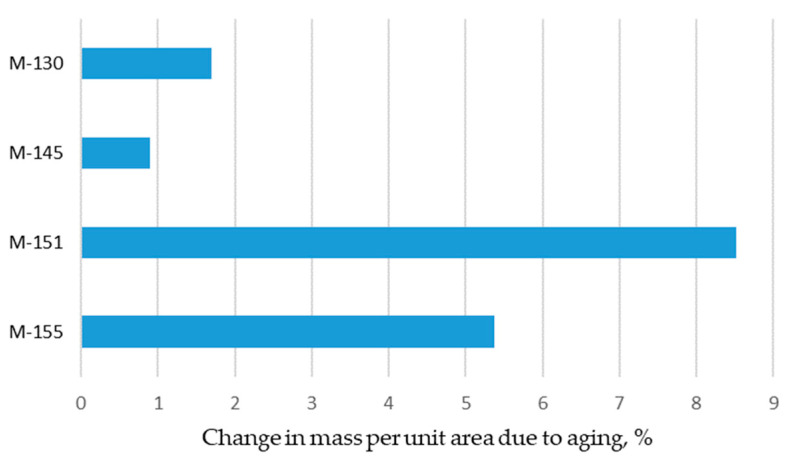
Changes in mass per unit area due to aging.

The results, which show the change in mass per unit area due to aging, are shown in Figure 2. The change in mass per unit area shown is compared to the mass per unit area values of the materials before aging. As can be seen from Figure 2, the mass per unit area increases after aging for all materials observed. The increase is up to 8.5% (the highest for the material labelled M-151). As can be seen, the increase in mass per unit area is higher for materials with lower declared masses, both for conventional and recycled materials. The previously discussed results also suggest that the aging process affects the shrinkage of the materials, as can be seen in the material property discussed previously.

### 3.3. Results of Material Density Testing

**Figure 3:**
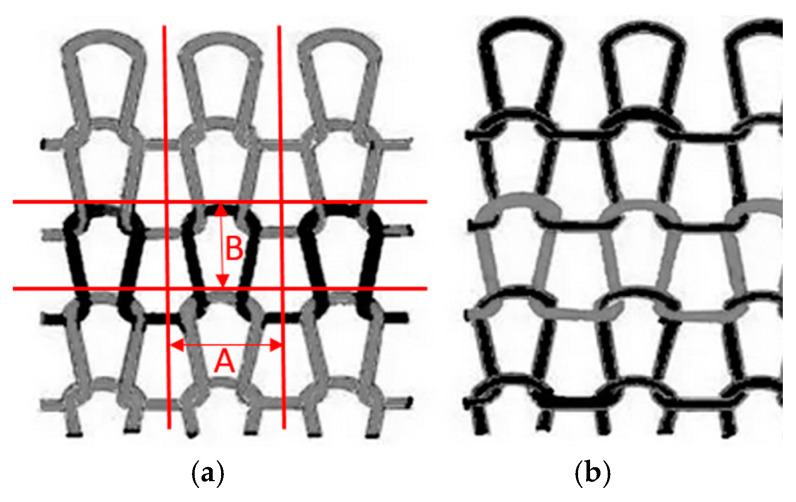
Structure model of knitted fabric: (**a**) Face; (**b**) Back.

In order to further explain the observations related to the outcomes of the material thickness and mass per unit area measurements, additional measurements of the horizontal (Dh) and vertical (Dv) densities were conducted. Figure 3 shows the structure model of knitted fabric including the fabric face (a) and back side (b). The Dh and Dv were determined by counting the numbers of segments A (wale spacing) and B (course spacing).

(**a**) 

(**b**)

**Figure 4:**
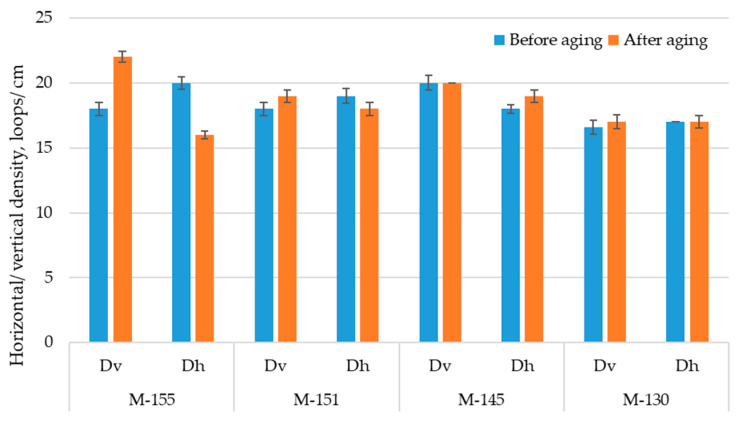
Horizontal (Dh) and vertical (Dv) densities of materials before and after aging.

As can be seen from Figure 4, the standard deviation of all measured densities are very low, in all cases less than 1 loop/cm.

The results show that the densities of the observed materials in the horizontal direction are different for tested materials and do not indicate the unequivocal behavior of an observed set of polyester materials in this direction. Opposed to that, there is an overall increase in the vertical density of materials that confirms the shrinkage of material in the vertical direction and is in line with the discussion related to the values of material thickness and mass per unit area. For these material properties, the mass of the material has a higher influence on the results after aging than the type of yarn (i.e., conventional vs. recycled). 

### 3.4. Results of Surface Roughness Testing

Table 3 shows the results obtained for surface roughness, with R_a_ being the selected index used to analyze the results of the surface roughness testing. The results include measurements in three directions, horizontal (direction shown in Figure 3), vertical and diagonal. The results show differences in the surface roughness between different materials and measurement directions, but they are not statistically significant (*p*-value was >0.05 in all cases). It is noticeable that the roughness is higher in the horizontal direction compared to the vertical direction, which could be explained by the structure of the main unit forming a structure of the material. In analyzing the materials before aging, sample M-130 and sample M-155 stood out. Sample M-130 showed the highest surface roughness values on the face side for all three tested measurement directions, which indicates that this material is rougher on the face side than the other tested samples. On the other hand, the M-155 sample showed the highest surface roughness values on the back side for all three test directions. After the aging process, the M-130 sample mostly showed lower roughness values, which means that the material became smoother after aging. In contrast, sample M-155 became rougher in certain directions after aging. 

During the aging of the material, variations in surface roughness may have resulted from the presence of small protruding fibers in the fabric structure. As the material ages, changes in these fibers, such as shifting or protruding, may lead to an increase in surface roughness. On the other hand, aging or use may cause some protruding fibers to retract or detach from the fabric, resulting in a smoother surface as irregularities causing roughness are removed. These processes depend on various factors, including the type of material and usage conditions, and a detailed analysis using a microscope allowed us to better understand the changes in surface roughness after the aging of the material, which can be seen in the figures in Table 2. After the aging process, a significant inverse proportional correlation was observed between the surface mass of the material (−0.94099) and the vertical density of the material (−0.89492) with the surface roughness on the back side, in the vertical direction. These results highlight the complexity of interdependencies among material properties after aging, emphasizing the need to consider various parameters to fully understand the mechanisms of changes in the structure and characteristics of the material. 

**Figure 5:**
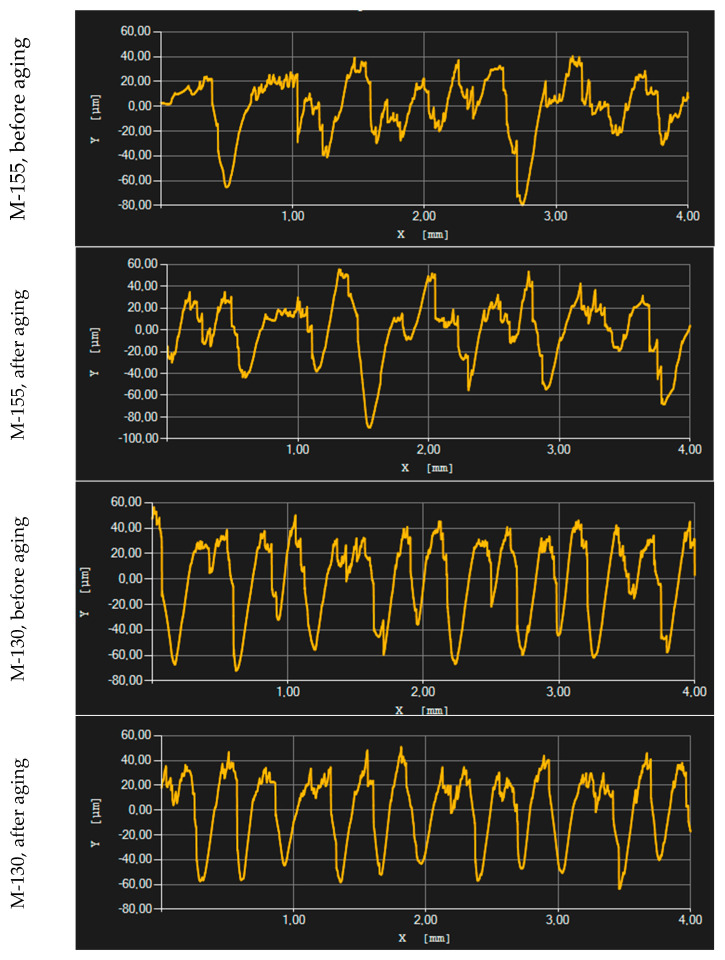
Characteristic illustration of R_a_ parameter results.

Figure 5 shows the graphs generated by the surface roughness measuring device. These graphs illustrate the height changes on the surface of the material versus distance. Specifically, the graphs show individual measurements in the horizontal direction for two different materials, M-155 and M-130, before and after the aging process. The vertical axis (Y) shows height changes on the surface of the material, while the horizontal axis (X) shows the measurement length or distance on the surface of the material. An analysis of the graphs reveals that differences between peaks and valleys on the material’s surface are evident for both samples, before and after the aging process. This indicates the presence of considerable irregularity or high surface roughness. After the aging process, material M-155 showed even greater height differences, resulting in increased roughness. This increase in surface roughness after the aging process suggests that this process had a detrimental impact on the surface quality of M-155, making it rougher and more uneven. Regarding material M-130, after the aging process, it showed slightly lower height differences. The reduced height differences could suggest that the aging process had a smoothing effect on M-130, leading to a decrease in surface roughness. 

Aging of football jersey materials, which is simulated to mimic long-term wear and tear, usually leads to unavoidable changes in material properties. The increase in roughness after this process can significantly negatively affect the performance of football players. Increased roughness can cause skin irritation, increase friction between the jersey and the skin and reduce comfort. A reduction in roughness, on the other hand, is not necessarily positive either, as it indicates a possible loss of important material properties, which can have a negative impact on the durability and structure of the jersey. Therefore, although aging may contribute to changes in roughness, these changes do not necessarily result in improved athletic performance, but often lead to a decrease in comfort and material quality. Moreover, the results of the previously conducted study [28] indicated that the perception of roughness is the most recognizable by the assessors, among a variety of material hand properties. 

### 3.5. Results of Tensile Testing

**Figure 6:**
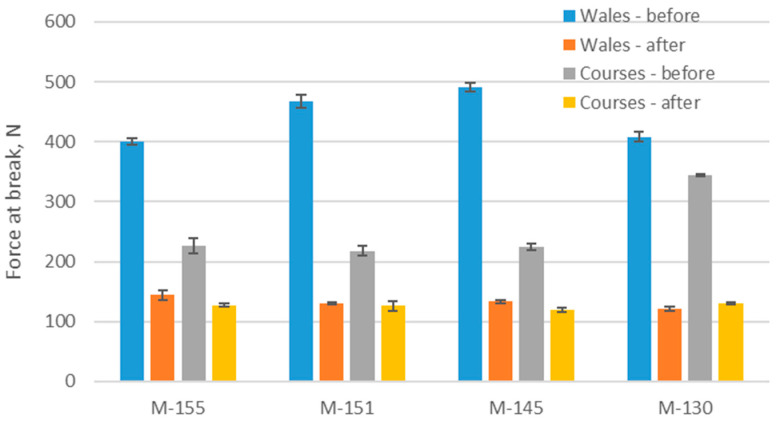
Forces at break of materials.

**Figure 7:**
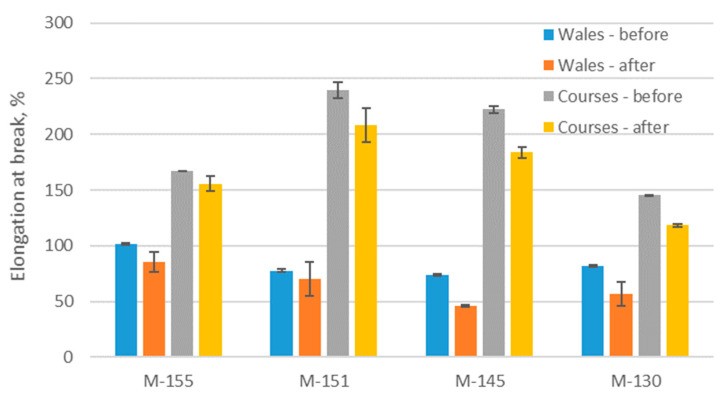
Elongations at break of materials.

**Figure 8:**
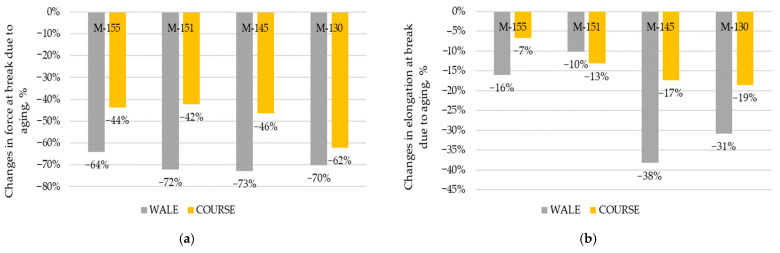
Changes due to aging (**a**) in force at break; (**b**) in elongation at break.

Figure 6 and Figure 7 show the results of force at break and elongation at break of the materials in the wale and course direction before and after the aging process. Figure 8 shows changes in force at break and elongation at break due to aging. The forces at break of the materials were usually higher in the wale direction due to a greater number of loops per unit length, which contribute to greater strength in that direction [49]. The standard deviations for force at break in the wale and course directions were very low, Figure 6. In analyzing the results of the force at break in the wale direction before aging, it can be seen that the values were between 400 and 490 N. After aging, these values were significantly reduced and were between 121 and 143 N, indicating a significant reduction in material strength in the wale direction after aging. There are statistically significant differences between forces at break in the wale direction (*p*-value is 0.0005). Sample M-145 showed the highest force at break in the wale direction before aging (490.79 N), but this value drastically decreased by 73% after aging, which can be seen in Figure 8a. On the other hand, sample M-155 had the lowest force at break in the wale direction before aging, and after aging, it recorded a decrease of 64%. In an earlier study [41], a decrease of up to 15% was found for fabrics composed of recycled polyester with shorter aging (corresponding to 24 sessions of active training and appropriate care). As shown in the results for the recycled polyester material (M-130), the longer aging time (40 sessions of active training) caused a striking decrease in the force at break, for 70%.

Regarding the force at break in the course direction before aging, the values were between 218 and 343 N, while after aging they were lower (between 120 and 130 N). Here again, a decrease in the strength of the material after aging is evident and is statistically significant (*p*-value is 0.01). Sample M-130 showed the highest force at break in the course direction before aging, but it also experienced a significant decrease of 62% after aging. On the other hand, sample M-151 had the lowest force at break in the course direction before aging and after aging, it recorded a decrease of 42%. These results indicate that the aging process significantly affected the strength of the material in both directions, reducing the forces at break after aging. Possible causes of these changes include fiber degradation, loss of elasticity, or changes in the internal structure of the material during the aging process. The highest average decrease in the forces at break for both directions studied was shown by a material composed of recycled yarn (−70% in the wale direction and −62% in the course direction), which makes this material the least recommended for use from this point of view. The observed decrease in the values of the forces at break after prolonged aging is in line with the observed decrease in bursting force reported in previously conducted study [39]. Still, the results presented in this study indicate that the decrease in the force at break is much more prominent when the aging of materials is prolonged. 

The elongation at break of a material is defined as the elongation of the sample corresponding to the breaking force of the material. The standard deviation for elongation at break was low. Only for sample M-151, it was more scattered after aging for both directions. From Figure 7, it can be seen how the elongation at break decreased after the aging process for both directions (wale and course). It is statistically significant for both directions (*p*-value for the wale direction is 0.01, and for the course direction, it is 0.009). The highest elongation at break in the direction of the course before aging was shown by sample M-151. However, after aging, the elongation at break of this sample decreased by 13%, as clearly seen in Figure 8b. On the other hand, sample M-145 showed the lowest elongation at break in the wale direction before aging and after the aging process, it decreased by 16%. As far as the elongation at break is concerned, the higher differences in values after aging are influenced by material mass rather than polymer type (conventional, recycled). The decreases in the elongations at break for materials M-155 and M-151 are similar to those previously reported for shorter periods of exposure [39], while the decrease in the same property for the material produced from recycled yarn is much higher. 

A decrease in the elongation at break may indicate a decrease in the durability of the material. During sport activities, materials are often exposed to different stresses. If materials lose their elasticity, they are more likely to be damaged or cracked. Ultimately, a reduction in elongation at break can negatively affect the functionality and comfort of sport apparel.

**Figure 9:**
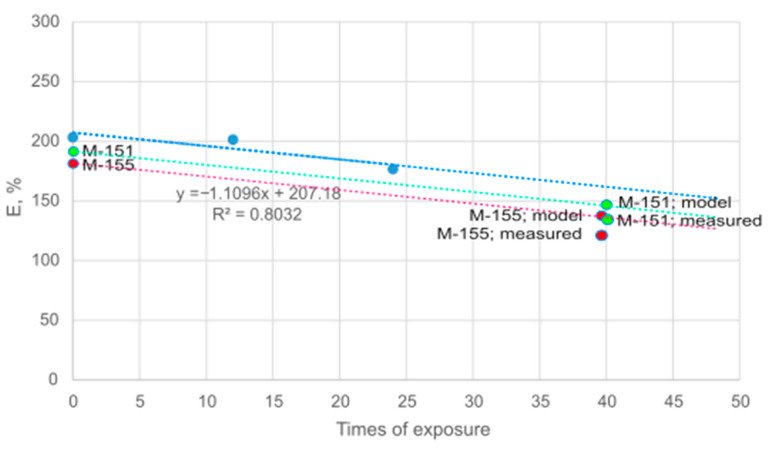
Comparison of elongation with a previously developed model presented in [39]: elongation of material tested and presented in [39] (in blue), elongation of material M-151 (in green) and elongation of material M-155 (in red).

The average values of elongation measured in this paper were compared with the model developed by a group of researchers in a previously published paper [39]. The model was created for the aging of a 100% polyester knitted material with a mass per unit area of 153 g/m^2^. It was developed for the elongation of the material after 12 and 24 simulated outdoor training sessions and had an R^2^ = 0.8032 (the results are presented in Figure 9 in blue). Materials M-155 and M-151, evaluated within this research, were used for comparison with the model, as both were also composed of 100% polyester and had similar masses per unit area, i.e., 155 and 151 g/m^2^, respectively (the results are presented in Figure 9 in red and green). The aging of materials M-155 and M-151 was converted to the number of times of exposure, which in this case was 40. As can be seen from Figure 9, the elongation of materials M-155 and M-151 were slightly lower than those predicted by the model, but both corresponded very well to the proposed model. 

### 3.6. Results of the Water Vapor Permeability Test

**Figure 10:**
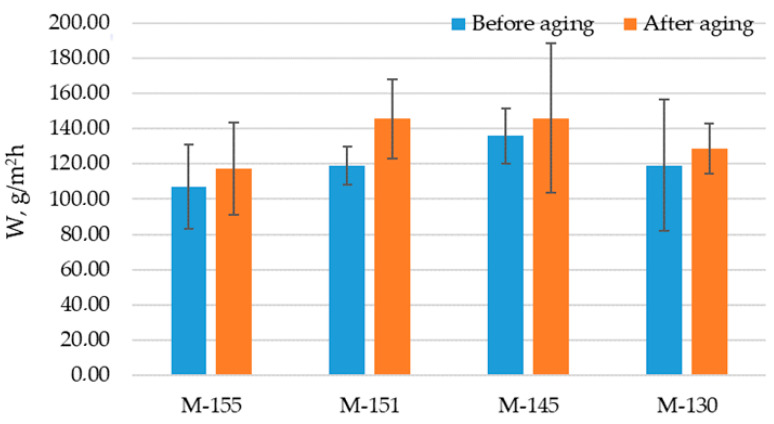
Water vapor permeability of the materials measured using PCE-MA device.

The results of water vapor permeability before aging ranged from 106.96 to 135.84 g/m^2^h (Figure 10). For the materials after aging, the results ranged from 117.36 to 145.84 g/m^2^h. The standard deviation depicted on the graph indicates increased variability in results after aging for samples M-155, M-151, and M-145. A significant dispersion in the data around the mean is clearly seen, suggesting higher variations or inhomogeneity’s in the material structure. These variations can arise from various factors, including the inhomogeneity of the material and differences in loop dimensions. Sample M-130 shows a reduced variability after aging, meaning that the data are less scattered around the mean value. In analyzing the results in Figure 10, it is seen that for all tested materials the results significantly increased after the aging process with a *p*-value of 0.02, which indicates a statistically significant difference between the mean values of the observed samples. Material M-145 stands out as the material with the highest water vapor permeability before and after aging. Before aging, the water vapor permeability for this material was 135.84 g/m^2^h, and then increased to 145.85 g/m^2^h after aging. The high water vapor permeability of this material makes it a favorable choice for active athletes due to the quick transfer of sweat, efficient regulation of body temperature and reduction of discomfort and skin irritation during intense physical activity. On the other hand, material M-155 showed the lowest water vapor permeability. This material also has the highest mass per unit among all tested samples. Therefore, it is not a suitable choice for sportswear intended for athletes engaged in intense workouts. 

The increase in water vapor permeability after aging can be explained as a result of various changes in the material’s structure during aging. For example, aging can lead to the opening of microscopic pores in the material or changes in the material’s surface structure [39,40], which would increase the material’s ability to transmit water vapor. To summarize the aging process leads to an increase in water vapor permeability and aging significantly affects this property of the materials.

### 3.7. Results of the Liquid Dispersion and Drying of Material 

**Figure 11:**
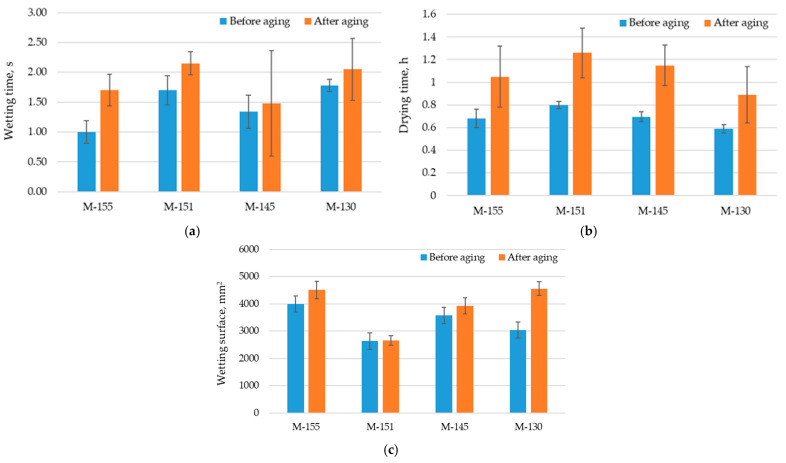
Liquid dispersion and drying of materials: (**a**) Wetting time; (**b**) Drying time; (**c**) Wetting surface.

The results shown in Figure 11a clearly indicate changes in wetting time before and after aging. It is evident that the materials require more time to absorb liquid after aging, and the statistically significant difference between the mean values of the observed samples confirms these changes (*p*-value is 0.02). The values for the wetting time before aging are between 1 s and 1.78 s, while after aging they are between 1.48 s and 2.15 s. Sample M-130 has the longest wetting time of 1.78 s before aging, while sample M-155 conversely requires the least time for liquid to penetrate the material (1 s). The aging process increased the wetting time for sample M-151 to 2.15 s, which had the longest wetting time of all the samples tested. It was observed that as the thickness of the knitted fabric increases after aging, the transfer of liquid through the knitted structure becomes more difficult. The increases in wetting time for materials after the aging process can be explained by a number of changes that occur in the material itself during this period. In observing the standard deviation depicted on the graph for wetting time, it can be noticed that the data are more scattered around the mean value for sample M-145 after aging. This may be a consequence of uneven aging of the material or changes in the sample’s structure, leading to diverse reactions or structural changes in the sample.

According to the results obtained for the wetting surface (shown in Figure 11c), an increase in the wetting surface was observed for all materials after aging. 

Despite this, the increase is not statistically significant (*p*-value is 0.07). Sample M-130 (recycled yarn) stood out as the material with the highest change in wetting surface. Before aging, this sample had a wetting surface of 3041 mm^2^, while the same sample had the largest wetting surface among all samples after aging (4557 mm^2^). On the other hand, sample M-151 had the smallest wetting surface after aging, which was 2651 mm^2^, compared to the initial value of 2631 mm^2^. This increase is significantly less than the one of sample M-130. The increase in wetting surface after aging can also be attributed to several factors, including material thickness and mass per unit area. These results indicate the complexity of the effects of aging on the physical properties of materials and demonstrate the importance of considering multiple parameters to fully understand the mechanisms of changes in the wetted surface. Although the statistical results may not be significant, the visual changes and variations between samples after aging encourage further investigations to better understand the factors contributing to these changes. The standard deviation depicted on the graph for wetting surface is less scattered around the mean value. 

Drying time is a critical factor in evaluating materials for sports. Materials with prolonged drying times can cause discomfort and are undesirable for this specific purpose [50]. As Figure 11b shows, the differences in drying times for all materials before and after aging are small but statistically significant (*p*-value is 0.001). The standard deviation depicted on the graph for drying time indicates increased variability in the results after aging. As previously mentioned, this may result from the uneven impact of aging on the material. Drying time before aging ranged from 0.59 to 0.80 h, while after aging, it ranged from 0.89 to 1.26 h. It is important to note that samples composed of conventional yarn had a longer drying time both before and after aging than a sample composed of recycled yarn. This statistically significant difference in drying times indicates the effects of aging on this important characteristic of the material. It is important to emphasize that the drying times of conventional yarns are also longer after aging, which may have an impact on the comfort and practicality of these materials in the context of sport equipment.

### 3.8. Results of the FTIR Analysis

**Figure 12:**
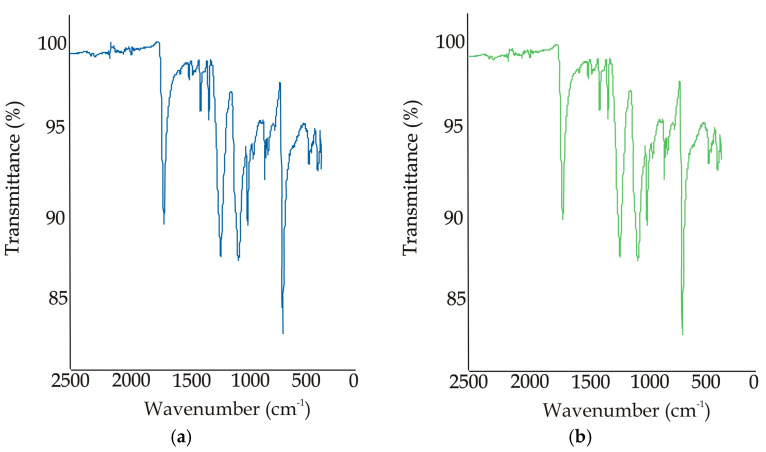
IR spectra: (**a**) before aging; (**b**) after aging.

The typical IR spectrum obtained for the investigated materials is shown in Figure 12. The characteristic absorption band in the spectrum (Figure 11a) is that of the stretching vibration of the carbonyl double bond C=O at 1712 cm^−1^. Absorption bands at 1239, 1093, 1016 and 722 cm^−1^ were detected in the fingerprint region (400–1400 cm^−1^). As can be seen from the spectra for the polyester material before aging (Figure 12a) and after aging (Figure 12b), the aging carried out in this study did not affect the chemical changes in the polymer. This applies to both the recycled and conventional polyesters.

## 4. Conclusions

This work highlighted the importance of studying the properties of the materials used for sportswear during their useful lifetime. The intention was to observe the changes in their properties due to aging, which can have a negative impact on the athlete’s performance.

The evidence from this study indicates that as the material ages, changes such as the shifting or protrusion of fibers from the material structure occur. These changes also affect the change in the surface roughness of the material. The surface roughness test showed that the aging process had a smoothing effect on the recycled polymer, resulting in a decrease in the roughness of the manufactured material. It was confirmed that aging leads to an increase in material thickness, especially for recycled polymer material, indicating a higher instability of the main structural unit. The highest average decrease in force at break for both directions studied was found in a material made of recycled yarn (−70% in the wale direction and −62% in the course direction), which makes this material the least recommended for use from this point of view. At the same time, a decrease in elongation at break of recycled material by up to 38% is expected to reduce the durability of the material compared to other materials tested. In terms of liquid management, the results have shown that the water vapour permeability of all polymeric materials increases after aging. This is a preferable behavior of the material as one of the main functions of sportswear material is to allow the release of perspiration from the body to the environment.

This study has highlighted the differences in the properties of polyester materials due to aging. In today’s competitive world, where athletic performance is paramount, the results of the study should be used to design even more functional materials that meet the needs of users.

## Figures and Tables

**Table 1:** Material description.

Material ID	Material Composition	Mass per Unit Area, g/m^2^	Yarn Fineness, Tex
M-155	100% PES (conventional yarn)	155	12
M-151	100% PES (conventional yarn)	151	10
M-145	100% PES (conventional yarn)	145	10
M-130	100% PES (recycled yarn)	130	10

**Table 2:** The surfaces of materials before and after aging.

	Material ID			
	M-155	M-151	M-145	M-130
Before aging	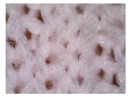	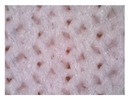	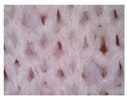	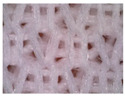
After aging	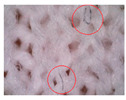	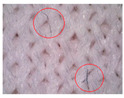	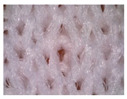	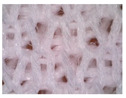

**Table 3:** Surface roughness (R_a_) of the material before and after aging.

		R_a_											
Materials ID		M-155			M-151			M-145			M-130		
		H	V	D	H	V	D	H	V	D	H	V	D
Before ageing	Face	18.468	9.105	19.768	15.238	8.645	15.870	16.184	8.769	16.058	26.392	12.462	21.750
	Back	20.774	10.483	21.292	15.508	10.404	13.778	17.220	9.995	13.836	20.094	10.072	19.032
After aging	Face	20.758	10.101	16.170	13.194	10.377	13.400	19.482	10.817	14.872	25.028	9.679	23.854
	Back	20.028	8.320	22.506	12.710	8.819	13.698	18.000	8.692	16.936	19.702	10.928	20.202

Legend: H—horizontally, V—vertically, D—diagonally.

## Data Availability

Data are contained within the article.
